# Mental state of central sterile supply department staff during COVID-19 epidemic and CART analysis

**DOI:** 10.1186/s12913-020-05864-5

**Published:** 2020-11-04

**Authors:** Wei Pan, Juan Hu, Liangying Yi

**Affiliations:** 1 Central Sterile Supply Department, West China Second University Hospital, Sichuan University/West China School of Nursing, Sichuan University, Chengdu, China; 2 Key Laboratory of Birth Defects and Related Diseases of Women and Children (Sichuan University), Ministry of Education, Chengdu, Sichuan China

**Keywords:** Central sterile supply department, Nurse, Logistic staff, Anxiety, Perceived stress, Resilience

## Abstract

**Background:**

During the COVID-19 epidemic, the central sterile supply department (CSSD) staff handled many devices, implements and non-disposable protective articles used by suspected or confirmed COVID-19 patients. As a result, the CSSD staff may have experienced psychological stress, however, the mental state of the CSSD staff during the COVID-19 epidemic has been rarely studied. We aim to investigate the mental state of the CSSD staff and relevant influencing factors experienced during the COVID-19 epidemic.

**Methods:**

The survey utilising a general information questionnaire, Chinese perceived stress scale (CPSS), self-rating anxiety scale (SAS), and Connor-Davidson resilience scale (CD-RISC) was conducted with 423 CSSD staff members from 35 hospitals in Sichuan Province, China. Data was analysed in SPSS24.0. Classification and regression tree (CART) was utilised to analyse variables and find variation between groups. A chi-square test was performed on enumeration data, and t-test and analysis of variance were performed on measurement data.

**Results:**

The CSSD staff’s SAS score was 37.39 ± 8.458, their CPSS score was 19.21 ± 7.265, and their CD-RISC score was 64.26 ± 15.129 (Tenacity factor score: 31.70 ± 8.066, Strength factor score: 21.60 ± 5.066, Optimism factor scores: 10.96 ± 3.189). The CPSS score was positively correlated with the SAS score (*r* = 0.66; *P* < 0.01), the CPSS score was negatively correlated with the CD-RISC score (*r* = − 0.617, *P* < 0.01), and the SAS score was negatively correlated with the CD-RISC score (*r* = − 0.477, *P* < 0.01). The job position, age, and political status of the CSSD staff were the main factors affecting their mental state; for example, the CPSS score and SAS score of the CSSD nurses were significantly different from those of the CSSD logistic staff (*P* < 0.01).

**Conclusion:**

During the epidemic, the CSSD staff’s psychological resilience was at a low level; the anxiety level of the CSSD nurses was higher than that of the CSSD logistic staff. Therefore, more attention should be given to the mental health of the CSSD staff, including taking protective measures regarding the risk factors to ensure they can maintain a healthy mental state.

## Background

In December 2019, several patients with novel coronavirus infection dominated by pulmonary lesions were observed in Wuhan, Hubei, China. In the document (NHC ML [2020] No. 42) issued by the National Health Commission of China on 7 February 2020, ‘Novel coronavirus infected pneumonia’ was temporarily named as ‘Novel coronavirus pneumonia’ [1]. On 11 February 2020, the World Health Organization announced Coronavirus Disease 2019 (simplified as ‘COVID-19’) as the name of the disease caused by 2019 novel coronavirus [2]. Before 11 March 2020, the authorities of 31 provinces (autonomous regions and central municipalities) and Xinjiang Production and Construction Corps in China reported a total of 16,145 confirmed cases of COVID-19 (including 4492 severe cases), 61,475 people discharged from the hospital after recovery, 3158 deaths and 14,607 close contacts still under medical observation [3].

For COVID-19, hospital-acquired infection is a factor that cannot be ignored [4]. The central sterile supply department, the key department for controlling hospital-acquired infections, assumed the important responsibility during the epidemic. Due to the special workplace and nature of the CSSD, its staff members are prone to occupational injury, which may have led to an increased psychological burden [5] during the epidemic. CSSD staff handled many devices, implements and non-disposable protective articles used by suspected or confirmed COVID-19 patients, therefore, close concern for CSSD staff members’ mental health is required. This study aims to investigate the mental state of the CSSD staff and relevant influencing factors experienced during the COVID-19 epidemic.

## Methods

### Participants

The nurses and logistic staff in the CSSDs of Secondary A or above hospitals in Sichuan Province, China who had more than 1 year first-line work experience in sterile supply and had not been absent from duty for 3 months or more were invited to participate in the study in February 2020. Also, interns were excluded from participating in this study. All the participants were informed of the purpose and significance of this study and voluntarily participated. Finally, data was gathered with convenience sampling.

### Survey tools

The survey utilising a general information questionnaire, Chinese perceived stress scale (CPSS), self-rating anxiety scale (SAS), and Connor-Davidson resilience scale (CD-RISC) was conducted with 423 CSSD staff members from 35 Secondary A or above hospitals in Sichuan Province, China.

The general information questionnaire was used to gather information about job position, gender, age, education background, hospital grade and political status from respondents. Ethical approval of this study was obtained from the Medical Ethics Committee of West China Second University Hospital, Sichuan University. Verbal consent was obtained from all study participants because this was investigation research, and it was conducted based on the online questionnaires which were voluntarily and anonymously completed by participants.

The Chinese Perceived Stress Scale (CPSS) was translated and modified by Yang and Huang [6] in 2003. The CPSS was used for evaluating the participant’s subjective stress perception level. The CPSS consisted of 14 items and analysed two factors, namely the sense of tension and the sense of vulnerability. The 5-point Likert scale was used, its score ranging from 1 to 5 assigned to the five response options: ‘never’, ‘seldom’, ‘sometimes’, ‘frequently’, ‘always’. The CPSS score was 14–70. Reverse scores were used with Items 4, 5, 6, 7, 9, 10 and 13; these seven items indicated the level of the participant’s sense of vulnerability. The sum of the scores of the remaining items was the score of the sense of tension. The sum of score of the sense of vulnerability and of the sense of tension was the CPSS score. When the CPSS score is higher than 25, it was judged that the participant was experiencing unhealthy levels of stress. The higher the CPSS score, the higher the perceived stress. Cronbach’s alpha for CPSS was 0.797.

The Self-rating Anxiety Scale (SAS), which was developed by Zung [7], was used for evaluating subjective perception of the participants who had anxiety symptoms, measuring the participants’ anxiety levels. It accurately reflected the participants’ subjective anxiety levels and provided reference for treatment. The SAS consisted of 20 items. The sum of the 20 items scores was the raw score. The index score was obtained by multiplying the raw score by 1.25 and keeping the integer. In this study, the classification was made based on the Chinese version of SAS in the Handbook of Common Psychological Evaluation Scales, modified by Dai [8], i.e. Index score < 50, No anxiety; Index score of 50–59, Mild anxiety; Index score of 60–69, Moderate anxiety; Index score of 69 or above, Severe anxiety.

The Connor-Davidson Resilience Scale (CD-RISC) was developed by US scholars Connor and Davidson. In this study, the Chinese version translated and modified by Yu and Zhang [9] was used. The Chinese version of CD-RISC consisted of 25 items and analysed 3 different factors, namely tenacity, strength and optimism. The 5-point Likert scale was used, with scores ranging from 0 to 4 that were assigned the following responses: ‘never’, ‘seldom’, ‘sometimes’, ‘frequently’, ‘always’. The CD-RISC score was 0–100. The higher the CD-RISC score, the higher the resilience. Cronbach’s alpha for CD-RISC was 0.91.

### Data collection

The electronic questionnaire was distributed via WJX, a professional online questionnaire system in Chinese. The CSSD nurses and CSSD logistic staff scanned a QR code to complete the questionnaire anonymously. The head nurse of the CSSD of each hospital supervised participants and reminded them to truthfully answer the questionnaires. All the questions in the questionnaire were set as required questions. A total of 423 questionnaires were distributed, and 423 valid questionnaires were returned. The valid recovery rate was 100%. The reliability of the questionnaire survey was analysed by SPSS version 24.0 and Cronbach’s alpha was 0.674.

### Statistical methods

Data from questionnaires was pre-processed to remove unfinished questionnaires. After data pre-processing, 423 samples and 101 variables were identified. A scatter diagram was used to study the correlations between CD-RISC, CPSS and SAS scores. The scatter diagram showed an obvious linear relationship between any two of the three variables. As all the variables of this study were nominal variables, CART, which is commonly used in sociology, was used for studying the influence of the social-demographic variables on the CPSS, SAS and CD-RISC scores. The Analysis of Variance (ANOVA) was used to evaluate the differences in mental state between the CSSD nurses and logistic staff. SPSS version 24.0 was used for data analysis. The enumeration data was described with the number and percentage, and the measurement data was described with the mean and standard deviation. The influencing factors of variables were examined with CART analysis. To find variations between groups, a chi-square test was performed on enumeration data, and t-test and ANOVA were performed on measurement data.

## Results

The general information acquired from CSSD staff members who participated in the study is presented in Table 1.

**Table 1:** General information of CSSD staff (*n* = 423)

Variable	n	%
**Gender**		
Male	45	10.63
Female	378	89.37
**Job Position**		
Nurse	335	79.2
Logistic Staff	88	20.8
**Age**		
18–25 years old	40	9.45
26–30 years old	48	11.35
31–40 years old	136	32.15
41–50 years old	151	35.7
>51 years old	48	11.35
**Educational Background**		
Junior High school and below	38	8.98
Senior high school / technical secondary school / technical school	61	14.42
Two or three years’ higher education diploma	162	38.3
Undergraduate	162	38.3
**Hospital Grade**		
Tertiary A	216	51.05
Tertiary B	112	26.48
Secondary A	95	22.46
**Political status**		
People without party affiliation	299	70.69
Members of the Chinese Communist Youth League	46	10.88
Probationary members of the Communist Party of China	5	1.18
Members of the Communist Party of China	63	14.89
Members of other parties	10	2.36

### CSSD staff’s CD-RISC score, CPSS score, SAS score and correlation

**Fig. 1:**
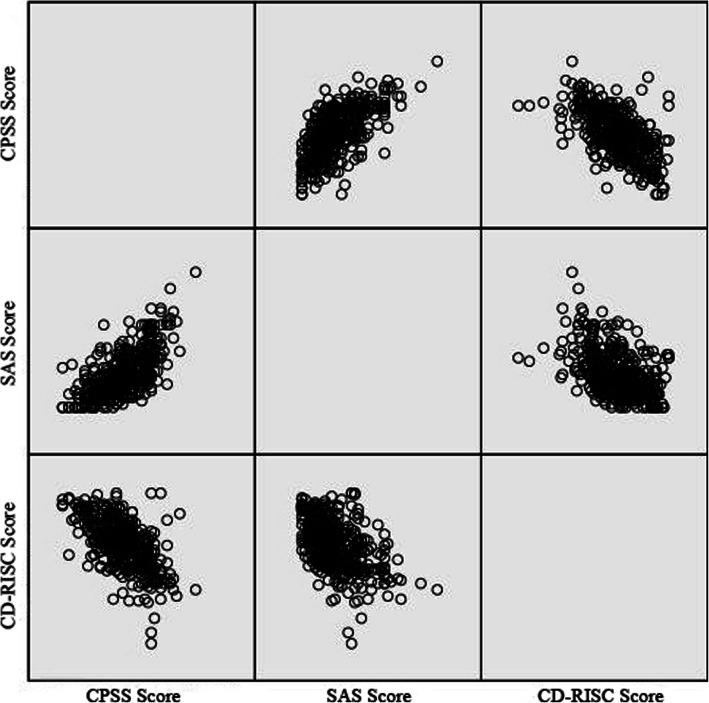
Scatter diagram for correlation between resilience, anxiety and perceived stress

The CSSD staff’s CD-RISC score was 64.26 ± 15.129 (Tenacity factor score: 31.70 ± 8.066, Strength factor score: 21.60 ± 5.066, Optimism factor scores: 10.96 ± 3.189), SAS score was 37.39 ± 8.458, and CPSS score was 19.21 ± 7.265. As revealed in Pearson correlation coefficient, the variables and the corresponding t-test result, the CSSD staff’s CPSS score was positively correlated with their SAS score (*r* = 0.66, *P* < 0.01), their CPSS score was negatively correlated with their CD-RISC score (*r* = − 0.617, *P* < 0.01); their SAS score was negatively correlated with their CD-RISC score (*r* = − 0.477, *P* < 0.01), as shown in Fig. 1.

### Influencing factors for resilience, perceived stress, and anxiety

**Fig. 2:**
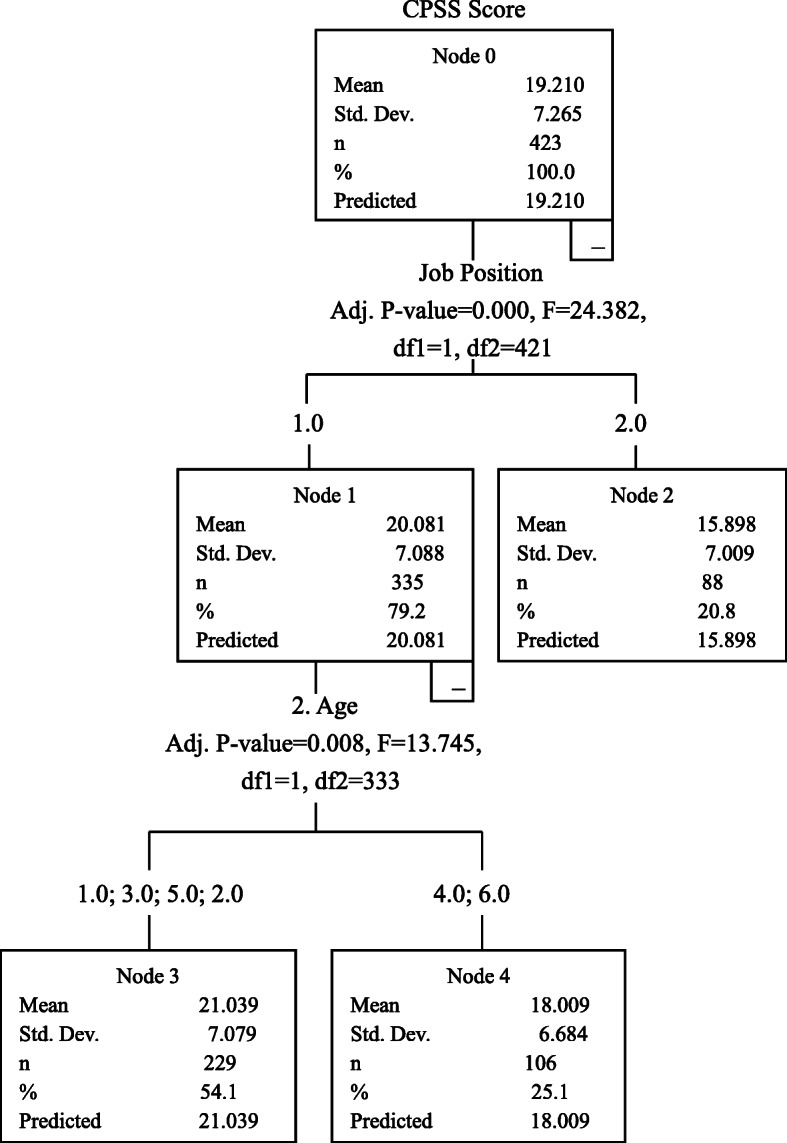
Influencing factors for CPSS score

**Fig. 3:**
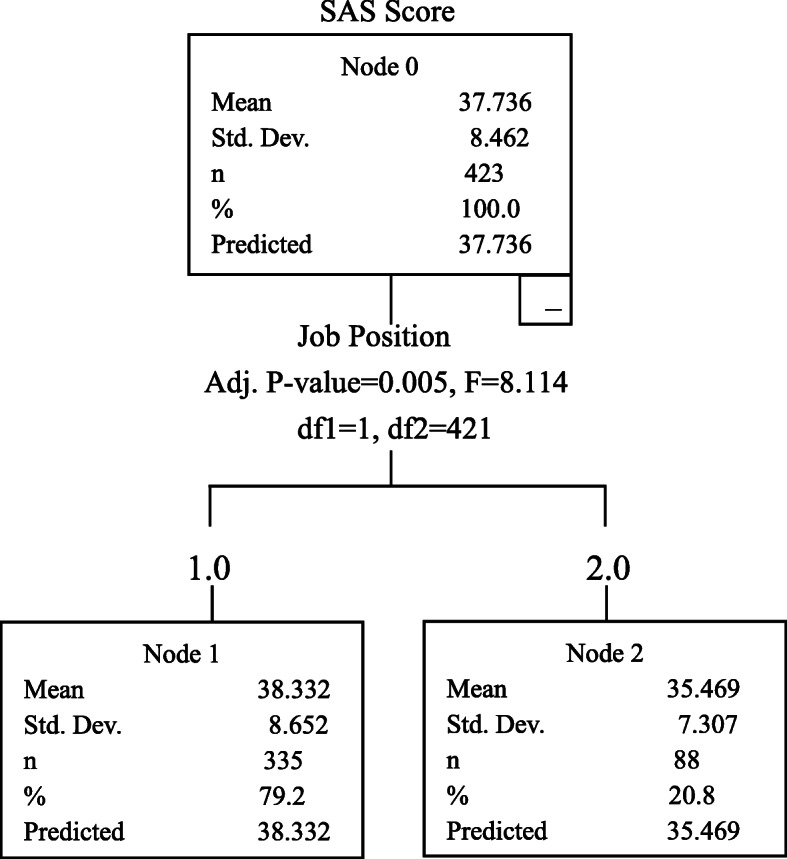
Influencing factors for SAS score

**Fig. 4:**
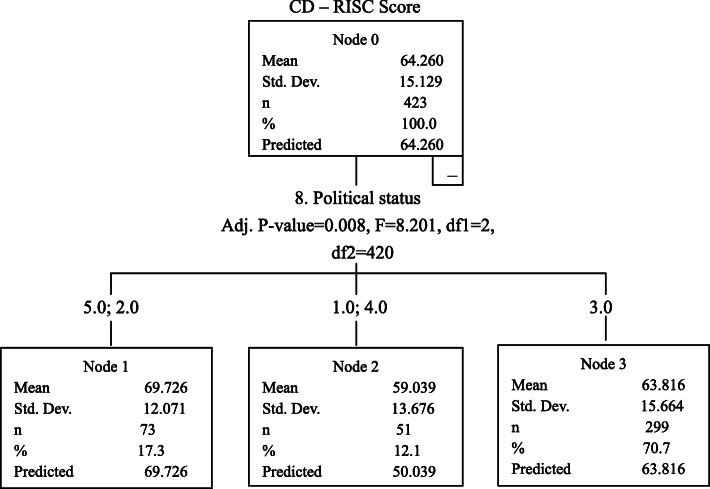
Influencing factors for CD-RISC score

CART analysis showed that job position and age of the CSSD staff were the two factors that had the most influence on perceived stress. The CPSS score (21.039 ± 7.079) of the CSSD nurses in the younger age groups (18–25, 26–30 and 31–40) was higher than the CPSS score (18.009 ± 6.684) of the CSSD nurses in the older age groups (41–50 and over 60); the CPSS score (15.898 ± 7.009) of the CSSD logistic staff was lower than that of the CSSD nurses. Job position was also a main factor influencing anxiety. The SAS score (38.332 ± 8.652) of the CSSD nurses was higher than the SAS score (35.469 ± 7.307) of the CSSD logistic staff. Political status was the main factor influencing the resilience. The CD-RISC score of members of the Communist Party of China and other parties was 69.726 ± 12.071, the CD-RISC score of people without party affiliation was 63.816 ± 15.664, and the CD-RISC score of members of the Chinese Communist Youth League and probationary members of the Communist Party of China was 59.039 ± 13.676. The CD-RISC score of members of the Communist Party of China and other parties was higher than that of the other two groups, and the mean scores for CD-RISC scores of these three groups were significantly different, as shown in Figs. 2, 3 and 4.

### One-way ANOVA for mental state

The analysis of differences in CPSS, SAS and CD-RISC scores pertaining to the CSSD nurses and the CSSD logistic staff was conducted with the one-way ANOVA. To compare variables, a chi-square test was performed on enumeration data. Also, a t-test between two groups of measurement data was performed as was an ANOVA between three or more groups of measurement data, as shown in Table 2.

**Table 2:** Analysis of variance for differences in mental state between CSSD nurses and CSSD logistic staff

		Type III sum of squares	df	Mean square	F value	*P* value
SAS Score	Between groups	571.39	1	571.39	8.114	0.005
	Within groups	29,645.594	421	70.417		
	Total	30,216.984	422			
CPSS Score	Between groups	1219.371	1	1219.371	24.382	0
	Within groups	21,054.903	421	50.012		
	Total	22,274.274	422			
CD–RISC Score	Between groups	152.568	1	152.568	0.666	0.415
	Within groups	96,434.827	421	229.061		
	Total	96,587.395	422			

## Discussion

### Analysis on mental state of CSSD staff

According to the survey results, the CPSS score of the CSSD staff was 19.21 ± 7.265, which was lower than that of the clinical nurses [10], but higher than that of community residents in China [11]. The perceived stress is the psychological response of the individual after perceiving and evaluating threatening stimuli in the environment [12]. The novel coronavirus chiefly transmits via respiratory droplets and close contact [13]; CSSD staff members were not in direct contact with the confirmed or suspected COVID-19 patients, therefore, CSSD staff had a lower perceived stress regarding the COVID-19 epidemic compared to clinical nurses. Due to the nature of work in the CSSD, the CSSD staff was at risk of being infected with the novel coronavirus through occupational exposure. Therefore, the CSSD staff had a higher perceived stress from the threat of the novel coronavirus compared to the community residents during the epidemic.

The survey results showed that the CD-RISC score of the CSSD staff was 64.26 ± 15.129, lower than the norm in China [14], meaning that the CSSD staff had less resilience. The survey results also showed that the optimism factor score was the lowest and the tenacity factor score was the highest. Because the novel coronavirus is transmitted quickly and widely, the number of confirmed and suspected cases increased continuously as it became an epidemic; the clinical workload and work pressure also increased [15], the CSSD staff had to handle a high workload while being at risk of infection from occupational exposure. During the early stages of the epidemic, protective articles were in shortage in China, particularly facial masks and protective gowns, thus the CSSD staff was unable to effectively cope with and adapt to the stress resulting from the emergent public health event. The CSSD staff with higher tenacity factor score had more control when facing stress and the emergent public health event, and were not easily influenced. Therefore, it is recommended that managers pay attention to the resilience level of the CSSD staff and provide them with specific mental support to improve their resilience and decrease the negative impact stemming from the epidemic.

According to the study results, the CSSD staff had a lower anxiety level. Their SAS score was 37.39 ± 8.458, lower than the threshold score of SAS, possibly because most of the participants were from Tertiary A hospitals and received strong support from the public and governments, along with protective measures, disease screening tools and treatment conditions. As a result, Tertiary A hospitals were able to meet employment demands. Also, most of the participants were of middle and upper level education backgrounds, they learned about the COVID-19 epidemic through proper channels and took effective measures to protect themselves.

### Analysis on correlation between CD-RISC, CPSS and SAS scores of CSSD staff

The survey results showed that the CSSD staff’s CPSS score was positively correlated with their SAS score (*r* = 0 .66, *P* < 0.01). That is to say, the higher the perceived stress, the more prevalent the anxiety. The possible reason is that the perceived stress was expressed as the tension and vulnerability of an individual, and the individual experienced negative moods, feelings, etc. to varying degrees [16]. As demonstrated by Rooij SRD, perceived stress increases to some extent when a person is in a state of depression or anxiety [17]. The findings are echoed by Wiegner, who found that perceived stress is usually accompanied by increased depression and/or anxiety [18]. The CSSD staff felt the negative pressure due to outbreak of coronavirus, and was prone to anxiety after working under such stressful conditions for extended periods of time. The CD-RISC score was negatively correlated with the CPSS score (*r* = − 0.617, *P* < 0.01), and the CD-RISC score was also negatively correlated with the SAS score (*r* = − 0.477, *P* < 0.01), which are basically consistent with the results of He, et al. [16] and Xie, et al. [19]. The scores indicate that the higher the resilience level, the lower the perceived stress and anxiety level. Individuals with higher resilience usually adopt optimistic and active attitudes under stressful conditions; they also know how to use external resources to handle problems [19]. During the epidemic, the higher the resilience, the higher the consciousness of self-protection. If the necessary protection measures are taken and the correct operation procedures are implemented, stress and anxiety can be relieved.

### Analysis on influencing factors for CPSS, SAS and CD-RISC scores

As shown in this study, job position and age of the CSSD staff were two factors that had high influence on the CPSS score. The CPSS score of the CSSD nurses was 20.081 ± 7.008, significantly higher than the CPSS score (15.898 ± 7.009) of the CSSD logistic staff; and the SAS score of the CSSD nurses was 38.332 ± 8.652, higher than the SAS score (35.469 ± 7.307) of the CSSD logistic staff. Due to the simple staffing structure in the CSSD, i.e. only the nurses and logistic staff in the CSSD, the CSSD logistic staff was responsible for handling most of the devices which required more physical labors, while the CSSD nurses played the leading role and were chiefly responsible for guidance and supervision of sterile supply. The CSSD nurses played an important role in controlling hospital-acquired infection. During the epidemic, controlling the hospital-acquired infection was the top priority in combating the epidemic, so the CSSD nurses had a higher level of perceived stress and anxiety compared to the CSSD logistic staff. Moreover, the CPSS score of the CSSD nurses in the younger age groups was higher than that of the CSSD nurses in the older age groups. The CSSD nurses in the younger age groups had shorter service periods, insufficient work experience and less experiences regarding hindrances; they also lacked strategies to use in response to stressful conditions [20]. Conversely, the CSSD nurses in the older age groups were very experienced in work and life, so these nurses could maintain a stable mood and had the necessary skills to respond to the emergent public health event.

The political status was a factor had the most influence on the CD-RISC score. The CD-RISC score of members of the Communist Party of China was 69.726 ± 12.071, higher than that of people without party affiliation (63.816 ± 15.664), and that of members of the Chinese Communist Youth League and probationary members of the Communist Party of China (59.039 ± 13.676). This is possibly because the members of the Communist Party of China always adhered to the mission of serving the public wholeheartedly, maintaining a spirit of contribution and perseverance.

## Conclusion

In summary, during the COVID-19 epidemic, the CSSD staff was under certain psychological stress, and more attention should be paid to the mental health of the CSSD staff. It is necessary to take specific measures to tackle the unhealthy mindset caused by the outbreak of the coronavirus, e.g. providing the CSSD staff more opportunities to learn new skills, organising emergency response drills and providing psychological counselling to improve their psychological resilience; keeping the team stable, and improving work quality and satisfaction. However, there might be some biases in this study due to the small sample size of the CSSD logistic staff. Further survey and studies are required to be performed in the future.

## Supplementary Information

**Additional file 1.** Questionnaire of perceived stress, anxiety and resilience of central sterile supply department staff during COVID-19 epidemic. The questions about general information of the participant, COVID-19 epidemic and mental state of participant could be found in the questionnaire. The questionnaires were used to research the mental state of CSSD staff during the COVID-19 epidemic.

## Data Availability

The datasets used and/or analysed during the current study are available from the corresponding author on reasonable request.

## Abbreviations

ANOVA: Analysis of variance
CART: Classification and regression tree
CD-RISC: Connor-Davidson resilience scale
COVID-19: Coronavirus Disease 2019
CPSS: Chinese perceived stress scale score
CSSD: Central sterile supply department
SAS: Self-rating anxiety scale
