# Supplementary material for: Mental state of central sterile supply department staff during COVID-19 epidemic and CART analysis
Source: BMC Health Serv Res. 2020 Nov 4;20:1006. doi: 10.1186/s12913-020-05864-5 (PMC7609829; doi:10.1186/s12913-020-05864-5)
Supplement: Supplementary file 1 — Additional file 1. Questionnaire of perceived stress, anxiety and resilience of central sterile supply department staff during COVID-19 epidemic. The questions about general information of the participant, COVID-19 epidemic and mental state of participant could be found in the questionnaire. The questionnaires were used to research the mental state of CSSD staff during the COVID-19 epidemic. [file 12913_2020_5864_MOESM1_ESM.docx]

**Questionnaire of Perceived Stress, Anxiety and Resilience of Central Sterile Supply Department Staff During COVID-19 Epidemic**

Dear Central Sterile Supply Department Staff,

Thank you very much for participating in the questionnaire survey. As an emergent public health event, the COVID-19 epidemic has brought enormous psychological stress to medical staff. Therefore, more attention shall be paid to mental health of medical staff in this difficult time. We are conducting a survey of perceived stress, anxiety and resilience. Please answer the following questions according to your actual situation in the past one month. Your answers are very important to our research. There are no right or wrong answers to these questions. Your answers will be kept confidential and will only be used for statistical analysis. Please feel free to answer these questions. Thank you for your cooperation.

**General Information**

1. What is your gender? [Single selection]*

| ○ Male |
| --- |
| ○ Female |

1. What is your age group? [Single selection]*

| ○Under 18 years old | ○18-25 years old | ○26-30 years old | ○31-40 years old | ○41-50 years old | ○51-60 years old | ○Over 60 years old |
| --- | --- | --- | --- | --- | --- | --- |

3. How many years of work experience do you have? [Single selection]*

| ○ Less than 1 year |
| --- |
| ○ 1-2 years |
| ○ 3-5 years |
| ○ 6-10 years |
| ○ 11-15 years |
| ○ 16~20 years |
| ○ Over 20 years |

4. Which Chinese ethnic group do you belong to? [Single selection]*

| ○ Han |
| --- |
| ○ Chinese ethnic minorities |

5. Where do you live? [Fill in the blank] *

_________________________________

6. What is your marital status? [Single selection] *

| ○ Never married |
| --- |
| ○ Married (have a child/children) |
| ○ Married (no child) |
| ○ Divorced |
| ○ Widowed |
| ○ Other _________________ |

7. Which of the following living situations applies to you? [Single selection] *

| ○ Live alone |
| --- |
| ○ Rent a room/apartment with other person(s) |
| ○ Live with family members |

8. What is your political status? [Single selection] *

| ○ Member of the Chinese Communist Youth League |
| --- |
| ○ Probationary member of the Communist Party of China |
| ○ Member of the Communist Party of China |
| ○ Member of other party |
| ○ Without party affiliation |

9. What is your educational background? [Single selection] *

| ○ Junior High school and below |
| --- |
| ○ Senior high school / technical secondary school / technical school |
| ○ Two or three years’ higher education diploma |
| ○ Undergraduate |
| ○ Master |
| ○ Doctoral |

10. What is the grade of the hospital you are working in? [Single selection] *

| ○ Tertiary A |
| --- |
| ○ Tertiary B |
| ○ Secondary A |
| ○ Secondary B |
| ○ Other _________________ |

11. What is your job title? [Single selection] *

| ○ Nurse |
| --- |
| ○ Logistic staff (Please go to Question 14) |

12. What is your professional title? [Single selection] *

| ○ Nurse |
| --- |
| ○ Senior nurse |
| ○ Supervisor nurse |
| ○ Co-chief nurse |
| ○ Chief nurse |

13. What is your position? [Single selection] *

| ○ Director of nursing |
| --- |
| ○ Associate director of nursing |
| ○ Head nurse |
| ○ Deputy head nurse |
| ○ None |

14. Which of the following staff categories applies to you? [Single selection] *

| ○ Intern |
| --- |
| ○ Staff taking the standardized training for residents |
| ○ Visiting staff |
| ○ Non-tenure-track employee |
| ○ Tenure-track employee |

**Questions about COVID-19 epidemic**

15. Have you ever contacted or met any confirmed COVID-19 patient in the past 14 days? [Single selection] *

| ○ Yes |
| --- |
| ○ No |

16. Have you ever contacted or met any suspected COVID-19 patient in the past 14 days? [Single selection] *

| ○ Yes |
| --- |
| ○ No |

17. Have you ever been to Wuhan, its surrounding areas or the place where there was a confirmed/suspected COVID-19 case in the past 14 days? [Single selection] *

| ○ Yes |
| --- |
| ○ No |

18. Is there a clustering of COVID-19 cases in your community? [Single selection] *

| ○ Yes |
| --- |
| ○ No |

19. What is your current situation? [Single selection] *

| ○ On vacation (Please go to Question 22) |
| --- |
| ○ Work (Please go to Question 22) |
| ○ Under combination of vacation and work (Please go to Question 22) |
| ○ Undergo home quarantine |
| ○ Undergo medical isolation |

20. How many days have you undergone home quarantine/medical isolation so far? [Please fill in the blank] *

_________________________________

21. Does other people’s attitude towards you change during your home quarantine or medical isolation? [Single selection] *

| ○ Yes, a very big change |
| --- |
| ○ Yes, a little bit of change |
| ○ No |

22. Have you stayed home except for working or buying daily necessities since the outbreak of COVID-19? [Single selection] *

| ○ Yes |
| --- |
| ○ No |

23. Do you always wear a mask when you go out? [Single selection] *

| ○ Yes |
| --- |
| ○ No |

24. Do you wash your hands carefully every day? [Single selection] *

| ○ Yes |
| --- |
| ○ No |

25. Do you take your temperature every day? [Single selection] *

| ○ Yes |
| --- |
| ○ No |

26. Do you have a cough or fever in recent days? [Single selection] *

| ○ Yes |
| --- |
| ○ No |

27. Does any of your family members have a cough or fever in recent days? [Single selection] *

| ○ Yes |
| --- |
| ○ No |

28. Has your hospital treated any confirmed/suspected COVID-19 patient? [Single selection] *

| ○ Yes |
| --- |
| ○ No |

29. Is your hospital a designated hospital for COVID-19 patients? [Single selection] *

| ○ Yes |
| --- |
| ○ No |

30. Have you ever handled the non-disposable devices, utensils or articles used by confirmed or suspected COVID-19 patients? [Single selection] *

| ○ Yes |
| --- |
| ○ No (Please go to Question 32) |

31.Which devices, utensils or articles have you handled? [Multiple selection] *

| □ Medical goggles |
| --- |
| □ Medical face shields and respirators |
| □ Surgical instruments, utensils and articles |
| □ Utensils for [anesthesia](C:/Users/sabaidee/AppData/Local/youdao/dict/Application/8.9.4.0/resultui/html/index.html#/javascript:;) |
| □ Other _________________ |

32. Does your hospital have enough medical protection resources? [Single selection] *

| ○ No, lack considerably |
| --- |
| ○ No, in short of resources |
| ○ Yes, adequate |
| ○ Yes, very abundant |

33. What are your job duties in recent days? [Multiple selection] *

| □ Collection |
| --- |
| □ Cleaning |
| □ Packaging |
| □ Sterilization |
| □ Distribution |
| □ Delivery |
| □ Other |

34. The protective measures you take for collecting the devices, utensils and articles used by confirmed or suspected COVID-19 patients include: [Multiple selection] *

| □ Workwear |
| --- |
| □ Disposable impermeable isolation gown |
| □ Surgical mask |
| □ Medical particulate respirator |
| □ Disposable medical cap |
| □ Medical goggles/face shield |
| □ Double latex gloves |
| □ Waterproof boots |

35. The protective measures you take for cleaning the devices, utensils and articles used by confirmed or suspected COVID-19 patients include: [Multiple selection] *

| □ Workwear |
| --- |
| □ Disposable impermeable isolation gown |
| □ Surgical mask |
| □ Medical particulate respirator |
| □ Disposable medical cap |
| □ Medical goggles/face shield |
| □ Double latex gloves |
| □ Waterproof boots |

36. The protective measures you take for packaging the devices, utensils and articles used by confirmed or suspected COVID-19 patients include: [Multiple selection] *

| □ Workwear |
| --- |
| □ Disposable impermeable isolation gown |
| □ Surgical mask |
| □ Medical particulate respirator |
| □ Disposable medical cap |
| □ Medical goggles/face shield |
| □ Double latex gloves |
| □Waterproof boots |

37. The protective measures you take for delivering the sterilized devices, utensils and articles used by confirmed or suspected COVID-19 patients include: [Multiple selection] *

| □ Workwear |
| --- |
| □ Disposable impermeable isolation gown |
| □ Surgical mask |
| □ Medical particulate respirator |
| □ Disposable medical cap |
| □ Medical goggles/face shield |
| □ Double latex gloves |
| □ Waterproof boots |

**Mental state (I)**

1. Are you anxious about anything unexpected? [Single selection] *

| ○ Never |
| --- |
| ○ Seldom |
| ○ Sometimes |
| ○ Frequently |
| ○ Always |

2. Do you feel that you are unable to control the important things in your daily life? [Single selection] *

| ○ Never |
| --- |
| ○ Seldom |
| ○ Sometimes |
| ○ Frequently |
| ○ Always |

3. Do you feel nervous and stressed? [Single selection] *

| ○ Never |
| --- |
| ○ Seldom |
| ○ Sometimes |
| ○ Frequently |
| ○ Always |

4. Can you successfully solve the problems that make you worry? [Single selection] *

| ○ Never |
| --- |
| ○ Seldom |
| ○ Sometimes |
| ○ Frequently |
| ○ Always |

5. Can you effectively deal with the important changes in your life? [Single selection] *

| ○ Never |
| --- |
| ○ Seldom |
| ○ Sometimes |
| ○ Frequently |
| ○ Always |

6. Are you confident of dealing with your own issues? [Single selection] *

| ○ Never |
| --- |
| ○ Seldom |
| ○ Sometimes |
| ○ Frequently |
| ○ Always |

7. Do things go smoothly as you wish? [Single selection] *

| ○ Never |
| --- |
| ○ Seldom |
| ○ Sometimes |
| ○ Frequently |
| ○ Always |

8. Are you unable to fulfill your own tasks? [Single selection] *

| ○ Never |
| --- |
| ○ Seldom |
| ○ Sometimes |
| ○ Frequently |
| ○ Always |

9. Are you able to deal with anything unpleasant? [Single selection] *

| ○ Never |
| --- |
| ○ Seldom |
| ○ Sometimes |
| ○ Frequently |
| ○ Always |

10. Are you in control of what happens in your life? [Single selection] *

| ○ Never |
| --- |
| ○ Seldom |
| ○ Sometimes |
| ○ Frequently |
| ○ Always |

11. Are you angry at anything that you can not control? [Single selection] *

| ○ Never |
| --- |
| ○ Seldom |
| ○ Sometimes |
| ○ Frequently |
| ○ Always |

12. Are you always thinking about anything that you must do? [Single selection] *

| ○ Never |
| --- |
| ○ Seldom |
| ○ Sometimes |
| ○ Frequently |
| ○ Always |

13. Can you control your own time? [Single selection] *

| ○ Never |
| --- |
| ○ Seldom |
| ○ Sometimes |
| ○ Frequently |
| ○ Always |

14. Do you feel that there are more and more problems but you can not solve them? [Single selection] *

| ○ Never |
| --- |
| ○ Seldom |
| ○ Sometimes |
| ○ Frequently |
| ○ Always |

**Mental State (II)**

1. Do you become more nervous and anxious than usual? [Single selection] *

| ○ Never/Seldom |
| --- |
| ○ Sometimes |
| ○ Frequently |
| ○ Usually/Always |

2. Do you feel frightened without a cause? [Single selection] *

| ○ Never/Seldom |
| --- |
| ○ Sometimes |
| ○ Frequently |
| ○ Usually/Always |

3. Do you get upset or panicky easily? [Single selection] *

| ○ Never/Seldom |
| --- |
| ○ Sometimes |
| ○ Frequently |
| ○ Usually/Always |

4. Do you feel that you are probably going crazy? [Single selection] *

| ○ Never/Seldom |
| --- |
| ○ Sometimes |
| ○ Frequently |
| ○ Usually/Always |

5. Do you feel that everything is OK and nothing bad will happen? [Single selection] *

| ○ Never/Seldom |
| --- |
| ○ Sometimes |
| ○ Frequently |
| ○ Usually/Always |

6. Have you ever experienced trembling in your arms and legs? [Single selection] *

| ○ Never/Seldom |
| --- |
| ○ Sometimes |
| ○ Frequently |
| ○ Usually/Always |

7. Have you ever suffered from headache, neck pain or backache? [Single selection] *

| ○ Never/Seldom |
| --- |
| ○ Sometimes |
| ○ Frequently |
| ○ Usually/Always |

8. Do you get weak or tired easily? [Single selection] *

| ○ Never/Seldom |
| --- |
| ○ Sometimes |
| ○ Frequently |
| ○ Usually/Always |

9. Do you feel calm and feel it easy to sit quietly? [Single selection] *

| ○ No/Seldom |
| --- |
| ○ Sometimes |
| ○ Frequently |
| ○ Usually/Always |

10. Does your heart beat fast? [Single selection] *

| ○ Never/Seldom |
| --- |
| ○ Sometimes |
| ○ Frequently |
| ○ Usually/Always |

11. Have you ever suffered from dizziness? [Single selection] *

| ○ Never/Seldom |
| --- |
| ○ Sometimes |
| ○ Frequently |
| ○ Usually/Always |

12. Have you ever fainted or felt faint? [Single selection] *

| ○ Never/Seldom |
| --- |
| ○ Sometimes |
| ○ Frequently |
| ○ Usually/Always |

13. Can you breathe easily and normally? [Single selection] *

| ○ Never/Seldom |
| --- |
| ○ Sometime |
| ○ Frequently |
| ○ Usually/Always |

1. Have you ever experienced numbness and tingling in your hands or feet? [Single selection] *

| ○ Never/Seldom |
| --- |
| ○ Sometimes |
| ○ Frequently |
| ○ Usually/Always |

15. Have you ever suffered from stomachache and indigestion? [Single selection] *

| ○ Never/Seldom |
| --- |
| ○ Sometimes |
| ○ Frequently |
| ○ Usually/Always |

16. Do you frequently feel the need to urinate? [Single selection] *

| ○ Never/Seldom |
| --- |
| ○ Sometimes |
| ○ Frequently |
| ○ Usually/Always |

17. Are your hands dry and warm? [Single selection] *

| ○ Never/Seldom |
| --- |
| ○Sometimes |
| ○ Frequently |
| ○ Usually/Always |

18. Does your face turn red and do you feel hot? [Single selection] *

| ○ Never/Seldom |
| --- |
| ○ Sometimes |
| ○ Frequently |
| ○ Usually/Always |

19. Do you fall asleep quickly and sleep well through the night? [Single selection] *

| ○ Never/Seldom |
| --- |
| ○ Sometimes |
| ○ Frequently |
| ○ Usually/Always |

20. Do you have nightmares? [Single selection] *

| ○ Never/Seldom |
| --- |
| ○ Sometimes |
| ○ Frequently |
| ○ Usually/Always |

**Mental State (III)**

1. Can you adapt to changes? [Single selection] *

| ○ Never |
| --- |
| ○ Seldom |
| ○ Sometimes |
| ○ Frequently |
| ○ Always |

2. Are you in an intimate and safe relationship? [Single selection] *

| ○ Never |
| --- |
| ○ Seldom |
| ○ Sometimes |
| ○ Frequently |
| ○ Always |

3. Are you proud of your achievements? [Single selection] *

| ○ Never |
| --- |
| ○ Seldom |
| ○ Sometimes |
| ○ Frequently |
| ○ Always |

4. Do you work hard in order to achieve your goals? [Single selection] *

| ○ Never |
| --- |
| ○ Seldom |
| ○ Sometimes |
| ○ Frequently |
| ○ Always |

5. Can you control your own life? [Single selection] *

| ○ Never |
| --- |
| ○ Seldom |
| ○ Sometimes |
| ○ Frequently |
| ○ Always |

6. Do you have a strong sense of purpose? [Single selection] *

| ○ Never |
| --- |
| ○ Seldom |
| ○ Sometimes |
| ○ Frequently |
| ○ Always |

1. Are you able to look on the positive side of things? [Single selection] *

| ○ Never |
| --- |
| ○ Seldom |
| ○ Sometimes |
| ○ Frequently |
| ○ Always |

8. Do you believe that everything happens for a reason? [Single selection] *

| ○ Never |
| --- |
| ○ Seldom |
| ○ Sometimes |
| ○ Frequently |
| ○ Always |

9. Do you have to do anything according to your feeling? [Single selection] *

| ○ Never |
| --- |
| ○ Seldom |
| ○ Sometimes |
| ○ Frequently |
| ○ Always |

10. Are you able to handle unpleasant emotions? [Single selection] *

| ○ Never |
| --- |
| ○ Seldom |
| ○ Sometimes |
| ○ Frequently |
| ○ Always |

11. Do you believe that sometimes God can help you? [Single selection] *

| ○ Never |
| --- |
| ○ Seldom |
| ○ Sometimes |
| ○ Frequently |
| ○ Always |

12. Are you able to deal with anything?  [Single selection] *

| ○ Never |
| --- |
| ○ Seldom |
| ○ Sometimes |
| ○ Frequently |
| ○ Always |

1. Does your previous success make you feel confident of overcoming challenges? [Single selection] *

| ○ Never |
| --- |
| ○ Seldom |
| ○ Sometimes |
| ○ Frequently |
| ○ Always |

1. Do you feel motivated under stress? [Single selection] *

| ○ Never |
| --- |
| ○ Seldom |
| ○ Sometimes |
| ○ Frequently |
| ○ Always |

15. Do you like challenges? [Single selection] *

| ○ Never |
| --- |
| ○ Seldom |
| ○ Sometimes |
| ○ Frequently |
| ○ Always |

16. Are you able to make an unusual or tough decision? [Single selection] *

| ○ Never |
| --- |
| ○ Seldom |
| ○ Sometimes |
| ○ Frequently |
| ○ Always |

17. Do you think you are a powerful person? [Single selection] *

| ○ Never |
| --- |
| ○ Seldom |
| ○ Sometimes |
| ○ Frequently |
| ○ Always |

18. Have you tried your best to do something regardless of the outcome? [Single selection] *

| ○ Never |
| --- |
| ○ Seldom |
| ○ Sometimes |
| ○ Frequently |
| ○ Always |

1. Can you achieve you goals? [Single selection] *

| ○ Never |
| --- |
| ○ Seldom |
| ○ Sometimes |
| ○ Frequently |
| ○ Always |

20. Have you even been discouraged by setbacks? [Single selection] *

| ○ Never |
| --- |
| ○ Seldom |
| ○ Sometimes |
| ○ Frequently |
| ○ Always |

1. Do you recover quickly from hardship or illness? [Single selection] *

| ○ Never |
| --- |
| ○ Seldom |
| ○ Sometimes |
| ○ Frequently |
| ○ Always |

22. Do you know where to seek help? [Single selection] *

| ○ Never |
| --- |
| ○ Seldom |
| ○ Sometimes |
| ○ Frequently |
| ○ Always |

23. Are you able to concentrate on what you are doing and think clearly under stress? [Single selection] *

| ○ Never |
| --- |
| ○ Seldom |
| ○ Sometimes |
| ○ Frequently |
| ○ Always |

24. Have you ever played a leading role in solving the problems? [Single selection] *

| ○ Never |
| --- |
| ○ Seldom |
| ○ Sometimes |
| ○ Frequently |
| ○ Always |

1. As long as you take adequate protective measures, you can prevent yourself from being infected by coronavirus. To what extent do you agree or disagree? [Single selection] *

| ○ Strongly agree |
| --- |
| ○ Agree |
| ○ Disagree |
| ○ Strongly disagree |

1. Provided that there are adequate protective measures, you are confident of handling the devices, utensils and articles used by confirmed or suspected COVID-19 patients. To what extent do you agree or disagree? [Single selection] *

| ○ Strongly agree |
| --- |
| ○ Agree |
| ○ Disagree |
| ○ Strongly disagree |

1. Do you know that the government has launched a helpline for the epidemic of novel coronavirus pneumonia? [Single selection] *

| ○ Yes |
| --- |
| ○ No |

1. What help do you urgently need? [Multiple selection] *

| □ Further information about novel coronavirus pneumonia |
| --- |
| □ More information about personal protective measures |
| □ Psychological counseling |
| □ Other _________________ |
